# Optimal Neutron Spectrum Database for In‐reactor ^238^Pu Production

**DOI:** 10.1002/advs.202410995

**Published:** 2025-01-28

**Authors:** Qingquan Pan, Xiaojing Liu, Yun Cai

**Affiliations:** ^1^ School of Nuclear Science and Engineering Shanghai Jiao Tong University Shanghai 200240 China; ^2^ Science and Technology on Reactor System Design Technology Laboratory Nuclear Power Institute of China Chengdu 610200 China

**Keywords:** ^238^Pu, genetic algorithm, nuclear batteries, point burnup, spectrum optimization

## Abstract

Plutonium‐238 (^238^Pu) is a scarce heat‐source radioisotope used in nuclear batteries, which is produced by in‐reactor irradiation of Americium‐241 (^241^Am) or Neptunium‐237 (^237^Np). Optimizing the neutron spectrum can improve the production efficiency of ^238^Pu, but currently, it is still a lack of knowledge about the optimal neutron spectrum for ^238^Pu production. Genetic algorithms and burnup algorithms are combined to identify optimal neutron spectra for ^238^Pu production under various irradiation times and flux levels, and build an optimal neutron spectrum database, which answers the questions “What is the optimal neutron spectrum for ^238^Pu production?” and “What is the maximum efficiency for ^238^Pu production” once and for all. This database can be referred to for determining the optimal neutron spectrum, guiding the neutron spectrum regulation process to improve the yield of ^238^Pu. Moreover, these results find that the production method of in‐reactor irradiation ^237^Np not only has a higher yield but also achieves a higher purity of ^238^Pu than that of in‐reactor irradiation ^241^Am, which conflicts with the traditional experiences, providing with previously unknown insights and helping break through the efficiency limit of traditional methods, maximizing ^238^Pu production.

## Graphical abstract

This database gives the first answer to “What is the optimal neutron spectrum for ^238^Pu production?” and “What is the maximum efficiency for ^238^Pu production?” once and for all. This database can be directly referred to for determining the optimal neutron irradiation spectrum, guiding the spectrum regulation process to improve the yield of ^238^Pu.

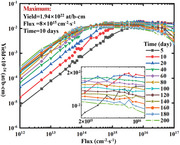

## 1 Introduction

Plutonium‐238 (^238^Pu) is a radioactive isotope prone to α‐decay, renowned for its efficient heat generation. Its released α‐particles can be easily shielded, and its decay daughter is Uranium‐234 (^234^U) which is a long‐lived nuclide,^[^ ^1^ ^]^ facilitating the elimination of radiation protection issues. Equation (1) gives the decay chain of ^238^Pu.

(1)

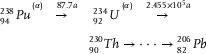




Therefore, ^238^Pu serves as heat source material owing to its unique properties.^[^ ^2^ ^]^ Notable examples include the heat source radioisotope thermoelectric generator used in the Galileo spacecraft^[^ ^3^ ^]^ and the isotopic pulse cardiac pacemakers.^[^ ^4^ ^]^

There are two established methods to produce ^238^Pu: 1) in‐reactor irradiation of Americium‐241 (^241^Am),^[^ ^5^ ^]^ as shown in Equation (2):

(2)

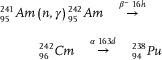

and 2) in‐reactor irradiation of Neptunium‐237 (^237^Np),^[^ ^6^ ^]^ as shown in Equation (3):

(3)

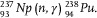




**Figure 1:**
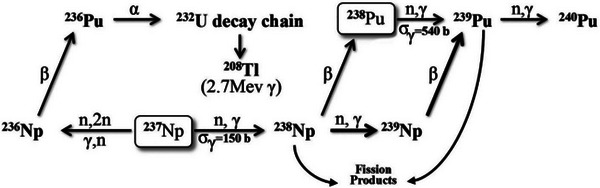
Nuclide transmutation path of in‐reactor irradiation of ^237^Np.

While the first method yields ^238^Pu with a higher purity in the traditional experiences, it generates intense gamma rays, degrading the radioactivity environment. Conversely, the second method poses almost no radioactivity issues, becoming the prevailing method for ^238^Pu production.^[^ ^7^ ^]^ Long‐term irradiation of ^237^Np targets leads to the emergence of numerous new nuclides and various nuclear reaction channels, which are coupled and form a complex nuclide transmutation process, as depicted in **Figure** **1**.

^238^Pu production faces challenges due to inefficient transmutation rates and high costs, exacerbated by inadequate neutronic models that failed to accurately simulate the complex nuclide transmutation process.^[^ ^8^, ^9^ ^]^ The microscopic cross‐section of nuclei in the transmutation path is related to the neutron energy spectrum.^[^ ^10^ ^]^ Within some energy regions, the nuclide chain is prone to reactions that facilitate the synthesis of ^238^Pu, enhancing the efficiency of ^238^Pu production and vice versa. Therefore, the efficiency can be improved by regulating the neutron irradiation spectrum, but the optimal spectrums for ^238^Pu production under various irradiation conditions are unknown.

Much research has been done to identify the optimal spectrum for ^238^Pu production. Notable examples include the rapid diagnosis method,^[^ ^11^ ^]^ the key nuclides analysis method,^[^ ^12^ ^]^ the filter burnup method,^[^ ^13^ ^]^ the single‐energy burnup method,^[^ ^14^ ^]^ the extreme burnup method,^[^ ^14^ ^]^ and Hogle's method^[^ ^15^, ^16^ ^]^ et al. These methods can quantify the values of neutrons across different energy regions for transuranium isotope production, but face two problems: 1) errors due to physical approximations, and 2) unknown of combining multiple energy regions into an optimal neutron spectrum. Therefore, we still lack knowledge about the optimal neutron irradiation spectrum for ^238^Pu production and its theoretical maximum yield under existing reactor conditions.

In this study, we identified optimal neutron irradiation spectra for ^238^Pu production under various irradiation times and flux levels and built an optimal neutron spectrum database, which answers the question “What is the optimal neutron irradiation spectrum for ^238^Pu production?” once and for all. This database can be directly referred to for determining the optimal neutron irradiation spectrum, guiding the spectrum regulation process to enhance efficiency and cost‐effectiveness. Meanwhile, the procedure for building a similar database can also be used for other isotopes, and the program we developed is available openly for a reader to build their database. The paper is structured as follows: Section 2 introduces the simulation method, Section 3 introduces the optimal neutron spectrum database of the two production methods, and Section 4 concludes.

## 2 Simulation Method

### 2.1 Point Burnup Method

We improve the universality of the optimal neutron spectrum database from two perspectives: 1) treat the target as a 0D point without considering the target's geometry,^[^ ^17^ ^]^ and 2) adopt a refined energy structure by dividing the totality into 238 energy regions.^[^ ^18^ ^]^

The point burnup method^[^ ^19^ ^]^ does not need to be coupled with the Monte Carlo method, having a high calculation efficiency to support the calculation of a large number of irradiation schemes. The Nuclear Library used is ENDF/B‐VII.1.^[^ ^10^ ^]^ The time‐dependent point burnup equation for each nuclide in the burnup chain is solved,

(4) $$\frac{dn_{i}}{dt}=\sum_{i\neq j}b_{j,i}^{\mathrm{eff}}\lambda_{j}^{\mathrm{eff}}n_{j}-\lambda_{i}^{\mathrm{eff}}n_{i}$$

where *n_i_* is the density of the *i*th nuclide, *λ_i_* ^eff^ is the effective decay constant of the *i*th nuclide, and *b_i_*_,_ *_j_* ^eff^ is the branching ratio for transmutating the *i* ^th^ nuclide to the *j* ^th^ nuclide. *λ_i_* ^eff^ and *b_i_*_,_ *_j_* ^eff^ can be calculated from the following formula,

(5) $$\left\{\begin{array}{c} \lambda_{i}^{\mathrm{eff}}=\lambda_{i}+\phi \sum_{j}\sigma_{i,j}\\ b_{i,j}^{\mathrm{eff}}=\left(b_{i,j}\lambda_{i}+\sigma_{i,j}\phi \right)/\lambda_{i}^{\mathrm{eff}} \end{array}\right.$$

where *λ_i_* is the decay constant of the *i*th nuclide, *ϕ* is the neutron flux, and *σ_i_*_,_ *_j_* is the one‐group cross‐section where the *i*th nuclide's reaction generates the *j*th nuclide.

We obtain a large number of neutron spectra by adjusting the percentages of neutron flux in these 238 energy regions. The one‐group cross‐sections of a neutron spectrum needed by the burnup calculation are calculated by

(6) $$\Sigma_{r}=\sum_{i=1}^{238}\sigma_{r,i}\cdot \phi_{i}$$

where the subscript “*i*” represents an energy region, subscript “*r*” represents a reaction type, *ϕ* represents the percentage of neutron flux, *σ* represents the grouped cross‐section.

### 2.2 Genetic Algorithm

As an intelligent optimization method, the genetic algorithm (GA) searches for possible solutions to problems by borrowing from the process of biological genetic evolution.^[^ ^20^ ^]^ Transform a specific problem into some specific chromosome coding strings to represent the individuals that make up the population, and each coding string is composed of several gene segments. These gene segments are crossed, mutated, and selected for the optimal solution. The basic operations of GA are as follows:

1. Initialization: Determine the evolutionary generations *G* and the population size *P*, and randomly produce *P* individuals to construct the initial population. *G =* 200 and *P* = 200 for constructing the optimal spectrum database in this paper;
2. Evaluation: Calculate the fitness of each individual in the population. The fitness in this paper is the yield of ^238^Pu, which is given by the point burnup calculation;
3. Selection: Select partial individuals with the highest fitness for crossover and mutation to produce the next generation. One hundred individuals with the highest fitness are selected to enter the next generation in this paper;
4. Crossover: Random exchange of several gene segments between two individuals to obtain two new individuals. The multi‐point crossover is selected with the crossover rate of 40% in this paper;
5. Mutation: Randomly modifying several gene segments in some individuals to obtain some new individuals. The mutation rate is 40% in this paper;
6. Iteration and termination: Repeat (2)‐(5) until *G* generations are completed.

We develop a program to support the above calculations, which first calls the GA module to construct a large number of neutron spectra, forming different irradiation schemes, and then calls the point burnup calculation module to calculate the yield of ^238^Pu of these irradiation schemes, providing fitness for GA. We searched for the optimal spectra with different irradiation times and flux levels, where 200 × 200 = 40 000 scenarios were evaluated in each case.

### 2.3 Irradiation Conditions

Irradiation times and flux levels significantly impact the efficiency of ^238^Pu production, and to enhance the universality of the optimal irradiation spectrum database, we have considered as many irradiation conditions as possible. The flux level of the research reactors is generally from 10^12^ to 10^16^ (cm^−2^⋅s^−1^), with a criticality time of fewer than 200 days. We considered 12 irradiation times of 5 days, 10 days, 20 days, 40 days, 60 days, 80 days, 100 days, 120 days, 140 days, 160 days, 180 days, 200 days. We considered 35 flux levels (cm^−2^⋅s^−1^) of 1 × 10^12^, 5 × 10^12^, 1 × 10^13^, 2 × 10^13^, 3 × 10^13^, 4 × 10^13^, 5 × 10^13^, 6 × 10^13^, 7 × 10^13^, 8 × 10^13^, 9 × 10^13^, 1 × 10^14^, 2 × 10^14^, 3 × 10^14^, 4 × 10^14^, 5 × 10^14^, 6 × 10^14^, 7 × 10^14^, 8 × 10^14^, 9 × 10^14^, 1 × 10^15^, 2 × 10^15^, 3 × 10^15^, 4 × 10^15^, 5 × 10^15^, 6 × 10^15^, 7 × 10^15^, 8 × 10^15^, 9 × 10^15^, 1 × 10^16^, 2 × 10^16^, 3 × 10^16^, 4 × 10^16^, 5 × 10^16^, 1 × 10^17^. We considered the two established methods for ^238^Pu production. Therefore, the optimal neutron irradiation spectra for 840 irradiation conditions (12 × 35 × 2 combinations) were identified.

For each isotope produced, we give its yield per unit volume (at/b‐cm) with the optimal neutron irradiation spectrum, as well as the global optimal neutron irradiation spectrum under different irradiation times and flux levels. However, due to the immense amount of data, with 420 irradiation conditions considered for each isotope and each neutron irradiation spectrum subdivided into 238 energy bins, we present these data by figures rather than tables. Meanwhile, the program developed for building this optimal neutron irradiation spectrum database is openly accessible, enabling interested readers to construct their databases under other irradiation conditions. Please refer to “Data Availability” in Acknowledgments for detailed data and the program.

## 3 Optimal Spectrum

### 3.1 In‐Reactor Irradiation of 241Am

**Figure 2:**
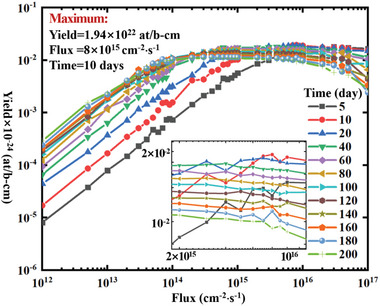
Yield of ^238^Pu under different irradiation conditions for the first method.

**Figure 3:**
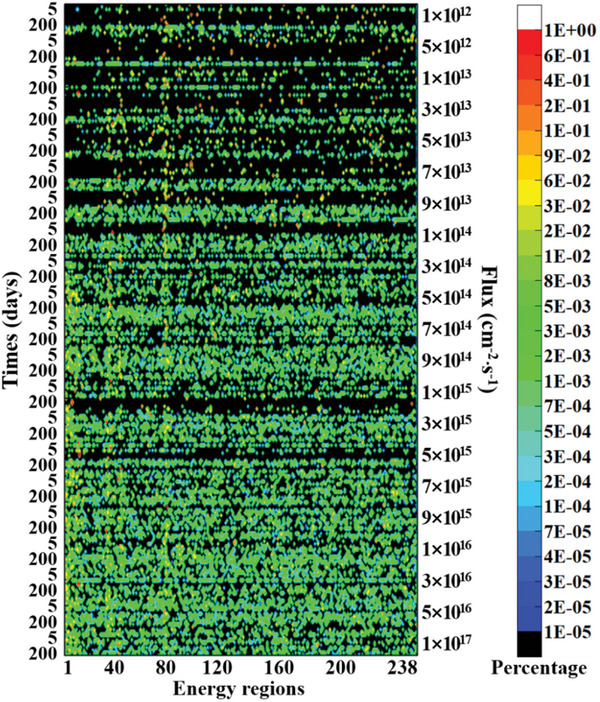
Optimal neutron irradiation spectra for ^238^Pu production for the first method.

The first method produces ^238^Pu by in‐reactor irradiation of americium dioxide (AmO_2_),^[^ ^21^ ^]^ and the nuclide components of the target are shown in **Table** **1**. The yields of ^238^Pu under different irradiation conditions for the first method are shown in **Figure** **2**. The optimal neutron irradiation spectra under different irradiation conditions for the first method are shown in **Figure** **3**.

**Table 1:** Nuclide components of the target for the first method.

isotopes	Number density [at/b‐cm]
^16^O	5.1104 × 10^22^
^241^Am	2.5527 × 10^22^
^242^Am	2.0430 × 10^17^
^243^Am	1.0624 × 10^19^

Among these 420 irradiation conditions, when the irradiation time is 10 days and the flux level is 8 × 10^15^ cm^−2^⋅s^−1^, the theoretical maximum yield of 1.94 × 10^22^ at/b‐cm can be reached for the first method. **Table** **2** gives the optimal neutron irradiation spectrum with the theoretical maximum yield of ^238^Pu for the first method.

**Table 2:** Global optimal neutron irradiation spectrum for the first method.

Energy Regions [MeV]	Percentages [%]
[1.25 × 10^−6^, 1.30 × 10^−6^]	11.00
[3.55 × 10^−5^, 3.70 × 10^−5^]	5.87
[7.50 × 10^−1^, 8.20 × 10^−1^]	22.74
[1.38 × 10^1^, 1.46 × 10^1^]	10.15
[1.73 × 10^1^, 2.00 × 10^1^]	50.25

### 3.2 In‐Reactor Irradiation of 237Np

**Figure 4:**
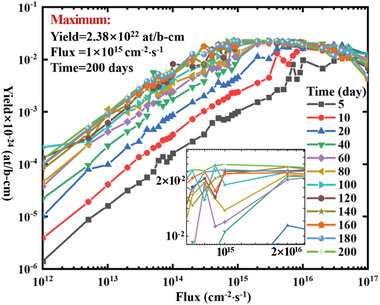
Yield of ^238^Pu under different irradiation conditions for the second method.

**Figure 5:**
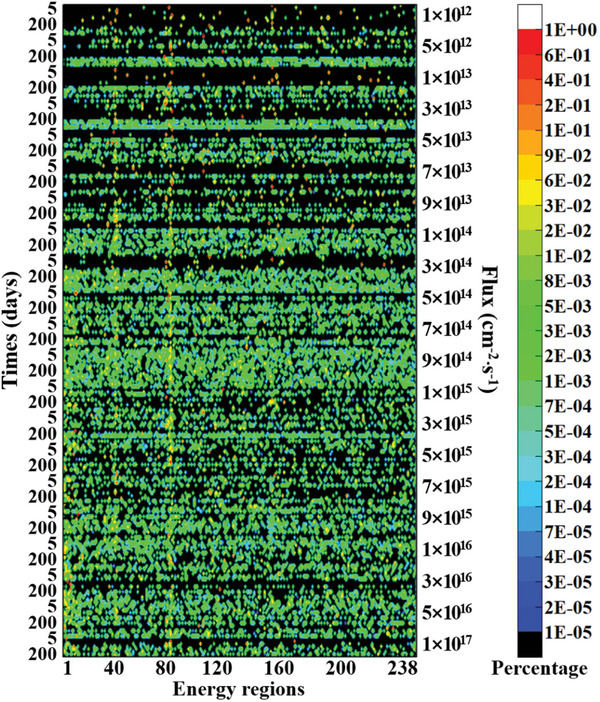
Optimal neutron irradiation spectra for ^238^Pu production for the second method.

The second method produces ^238^Pu by in‐reactor irradiation of neptunium dioxide (NpO_2_),^[^ ^6^ ^]^ and the nuclide components of the target are shown in Table 3. The yields of ^238^Pu under different irradiation conditions for the second method are shown in **Figure** **4**. The optimal neutron irradiation spectra under different irradiation conditions for the second method are shown in **Figure** **5**.

**Table 3:** Nuclide components of the target for the second method.

isotopes	Number density [at/b‐cm]
^16^O	4.9700 × 10^22^
^237^Np	2.485 × 10^22^

Among these 420 irradiation conditions, when the irradiation time is 200 days and the flux level is 1 × 10^15^ cm^−2^⋅s^−1^, the theoretical maximum yield of 2.38 × 10^22^ at/b‐cm can be reached for the second method. **Table** **4** gives the optimal neutron spectrum with the theoretical maximum yield of ^238^Pu for the second method.

**Table 4:** Global optimal neutron irradiation spectrum for the second method.

Energy Regions [MeV]	Percentages [%]
[4.92 × 10^−5^, 5.06 × 10^−5^]	69.04
[2.48 × 10^0^, 3.00 × 10^0^]	8.87
[1.73 × 10^1^, 2.00 × 10^1^]	22.09

### 3.3 Comparison of the Two Methods

As shown in Figures 2 and 4, when the flux is <1 × 10^15^ cm^−2^⋅s^−1^, both production methods follow the pattern that the higher the flux and the longer the irradiation time, the higher the yield of ^238^Pu. However, this pattern no longer holds when the flux is >1 × 10^15^ cm^−2^⋅s^−1^. Instead, an optimal irradiation time exists for a specific flux level. Further irradiation does not increase yield and may even reduce production, indicating the presence of over‐irradiation. Over‐irradiation refers to the point during irradiation when the maximum yield has been produced. If irradiation continues beyond this point, the yield of ^238^Pu will decrease due to nuclear reactions. The issue of over‐irradiation can significantly reduce the production efficiency of ^238^Pu. Therefore, the phenomenon of over‐irradiation should be considered during the production process, and the optimal irradiation time should be selected.

By comparing Figures 2 and 4, it can be seen that the theoretical maximum yield of ^238^Pu by in‐reactor irradiation of ^237^Np is slightly higher than that of in‐reactor irradiation of ^241^Am, but the difference is not significant. In addition, we have provided the yields of the isotopes of plutonium, as shown in **Table** **5**.

**Table 5:** Yields of the isotopes of plutonium.

Isotopes	Irradiation of ^241^Am	Irradiation of ^237^Np
^236^Pu	5.9213 × 10^16^	3.3711 × 10^18^
^237^Pu	6.9557 × 10^16^	1.5309 × 10^18^
^238^Pu	1.9414 × 10^22^	2.3832 × 10^22^
^239^Pu	2.3807 × 10^18^	1.1889 × 10^19^
^240^Pu	4.5931 × 10^18^	1.9682 × 10^19^
^241^Pu	5.4066 × 10^18^	2.7636 × 10^16^
^242^Pu	4.1793 × 10^21^	2.3884 × 10^14^
^243^Pu	8.9211 × 10^17^	4.9180 × 10^9^
^244^Pu	2.3138 × 10^16^	1.8984 × 10^8^
^245^Pu	3.9871 × 10^11^	3.3031 × 10^2^
^246^Pu	3.0593 × 10^9^	6.5039 × 10^0^
Proportion of ^238^Pu	82.240%	99.847%

As shown in Table 5, the second production method of in‐reactor irradiation of ^237^Np not only has a higher yield but also achieves a higher purity of ^238^Pu, having greater potential. However, the traditional ideas believe that the first production method of in‐reactor irradiation of ^241^Am can obtain ^238^Pu with higher purity.^[^ ^22^ ^]^ Therefore, the conclusion obtained in this paper is contrary to the traditional ideas. The authors regard this conflicting conclusion as the academic manifestation of this paper. That is, through our theoretical research, we can get rid of traditional experience, construct a more efficient production strategy, and further break through the efficiency limit of traditional methods.

In addition, as shown in Figures 2 and 4, the optimal neutron spectrum for both production methods is not continuous but a combination of fluxes in several energy bins, which is difficult to achieve by spectrum regulation. Despite the difficulty, we now have a direction for spectrum regulation, striving to make the neutron irradiation spectrum closer to the optimal one, where the closer to the optimal one, the higher the yield. These results present a previously unknown conclusion, helping us understand the process intuitively. Therefore, the research in this paper not only answers unknown questions but also provides theoretical support for subsequent engineering design, demonstrating its academic value.

## 4 Conclusion

Plutonium‐238 (^238^Pu) is a scarce heat‐source radioisotope produced by in‐reactor irradiation of Americium‐241 (^241^Am) or Neptunium‐237 (^237^Np), which involve complex nuclei transmutation. ^238^Pu production is limited by inadequate neutronic models to accurately simulate the complex nuclide transmutation process, facing challenges of inefficient transmutation rates and high costs. Theoretically, the production efficiency can be improved by regulating the neutron spectrum in the irradiation channel, but the optimal spectrums for ^238^Pu production under various irradiation conditions are unknown.

We identify the optimal neutron irradiation spectra for ^238^Pu production under different irradiation times and flux levels. The flux levels from 1.0 × 10^12^ to 1.0 × 10^17^ cm^−2^⋅s^−1^ and the irradiation times from 5 to 200 days are considered, covering the main irradiation conditions of reactors. An optimal neutron spectrum database is built, which answers the question “What is the optimal neutron spectrum for ^238^Pu production?” once and for all. This database can be directly referred to for determining the optimal irradiation spectrum for ^238^Pu production, showing the theoretical maximum yield of ^238^Pu and providing theoretical support for spectrum optimization.

We can also draw the following conclusions from this database: 1) the production method of in‐reactor irradiation of ^237^Np not only has a higher yield but also achieves a higher purity of ^238^Pu than that of the production method of in‐reactor irradiation of ^241^Am, which conflicts with the traditional experiences, helping us break through the efficiency limit of traditional methods, 2) there is over‐irradiation during ^238^Pu production, so this mechanism should be considered during the production process, and 3) the optimal neutron spectrum is not continuous but a combination of fluxes in several energy bins, which is difficult to achieve by spectrum regulation. Despite the difficulty, we now have a direction for spectrum regulation, striving to make the irradiation spectrum closer to the optimal one. These results present a previously unknown conclusion, helping us understand the process intuitively.

## Conflict of Interest

The authors declare no conflict of interest.

## Author Contributions

Q.P. and X.L.contributed to the writing, concept, calculation, analysis, and supervision. Y.C. contributed to concept, review and funding.

## Data Availability

All data can be available through the Link: https://pan.baidu.com/s/1LggW5xdxZbhBzbGIInyaew with the code “QPAN”.
